# University College Hospital

**Published:** 1902-03-29

**Authors:** 


### UNIVERSITY COLLEGE HOSPITAL.

The first meeting in the new board-room on Thursday, the 20th inst., was the "annual general meeting of governors, donors, and subscribers." Lord Monkswell, treasurer, was in the chair, and only twelve gentlemen gathered round the table "to elect by ballot fourteen members of the hospital committee, to receive a statement of accounts for the past year, and a report on the condition of the hospital."

The conduct of the meeting was purely formal, and it was all over within fifteen minutes of the hour announced. The Chairman went over the main points in the report, which was adopted unanimously. The new building, Lord Monkswell said, was making satisfactory progress. He was sorry to have to point out that a very considerable increase had occurred in the expenditure on maintenance. It would be a great calamity if the building had to be stopped.

Lack of Local Support.

Mr. Henry Lucas, chairman of the hospital committee, also called attention to the question of maintenance. The expenditure, he said, would increase year by year until the whole hospital was opened. The ordinary income from in vestments, annual subscriptions, donations, and grants from the central funds was only £14,000, so that were it not for the legacies, the committee would have been obliged to close a large portion of the hospital. Last year they were fortunate in receiving £13,000 in legacies, and that enabled them to meet the deficiency between the items of income and expenditure; but these receipts were far beyond the usual amount received in legacies—which was sometimes only one or two thousand pounds. They could not expect in future years to derive such a large amount from that source, and as a matter of fact the legacies ought to be invested. The fault lay to a great extent in the district, and the support afforded by it was utterly inadequate. Of the total amount of subscriptions (£2,400) and donations (£3,000), but a very small proportion came from the district. The new hospital had been erected by Sir John Blundell-Maple in recognition of the business he had done in the district, and the inhabitants should show their gratitude to him, apart from the duty they owed to the poor. They ought to remember that but for this hospital the rates would be higher. He would give two instances of the inadequacy of local support. There were 270 houses in Tottenham Court Road, all occupied by business people. Only 14 or 16 persons were subscribers. In Gower Street there were only six subscribers. If he were to go through all the streets it would be the same thing. He hoped the people in the district would recognise the benefit conferred by the hospital, and its duty towards maintaining it. If not, the committee would have to do as they did some years ago, and close a portion of the hospital. It was impossible to maintain it unless the necessary funds were forthcoming.

In reply to the Chairman's remark that the committee were anxious to keep the expenditure as low as possible, and even to diminish it, Mr. Lucas said, "I am afraid there cannot be much diminution, because the hospital now occupies a double amount of space."

The usual votes of thanks ended the meeting.

